# Voided Urinary Microbiota Is Stable Over Time but Impacted by Post Void Storage

**DOI:** 10.3389/fcimb.2020.00435

**Published:** 2020-08-25

**Authors:** Caspar Bundgaard-Nielsen, Nadia Ammitzbøll, Yusuf Abdi Isse, Abdisalam Muqtar, Ann-Maria Jensen, Peter D. C. Leutscher, Louise Thomsen Schmidt Arenholt, Søren Hagstrøm, Suzette Sørensen

**Affiliations:** ^1^Centre for Clinical Research, North Denmark Regional Hospital, Hjørring, Denmark; ^2^Department of Clinical Medicine, Aalborg University, Aalborg, Denmark; ^3^Department of Health Science and Technology, Aalborg University, Aalborg, Denmark; ^4^Steno Diabetes Center North Jutland, Aalborg, Denmark; ^5^Department of Gynecology and Obstetrics, North Denmark Regional Hospital, Hjørring, Denmark; ^6^Department of Pediatrics, Aalborg University Hospital, Aalborg, Denmark

**Keywords:** bacteria, DNA extraction, microbiome, microbiota, specimen handling, urine, urine storage

## Abstract

**Background:** New sensitive techniques have revealed a thriving bacterial community in the human urinary tract, challenging the perception that urine in healthy humans is sterile. While the functional role of this urinary microbiota is unknown, dysbiosis has been linked to urgency urinary incontinence and risk of urinary tract infections. When comparing studies, it is crucial to account for possible confounders introduced due to methodological differences. Here we investigated whether collection and storage conditions had any impact on the urinary microbial composition.

**Results:** For comparison of different storage conditions, midstream urine was collected from five healthy adult female donors and analyzed by 16S rRNA gene sequencing. Samples stored at −80 and −20°C, but not 4°C, were found to be comparable to freshly handled voided urine. Using the same methods, the daily or day-to-day variation in urinary microbiota was investigated in 19 healthy donors, including four women, five men, five girls, and five boys. Apart from two male adult donors, none of the tested conditions gave rise to significant differences in alpha and beta diversities between individuals.

**Conclusion:** The composition of voided urinary microbiota was found to be effectively maintained by freezing, but not storage at 4°C. In addition, we did not observe any intrapersonal daily or day-to-day variations in microbiota composition in women, girls or boys. Together our study supports present methodologies that can be used in future studies investigating the urinary microbiota.

## Background

It has been established that the human body has a symbiotic relationship with an abundance of microorganisms, which play a role in maintenance of health. In particular, microorganisms present in the gut have received much attention, and many studies have described their beneficial functions in immune regulation (Chung et al., 2012; Rosser et al., 2014) and metabolic processes (Velagapudi et al., 2010). However, when brought out of balance (dysbiosis), the same microorganisms have been associated with several pathological states including infections (Chang et al., 2008), autoimmune diseases (Pedersen et al., 2016), obesity (Turnbaugh et al., 2009), and psychiatric or neurodevelopmental disorders (Finegold et al., 2010; Jiang et al., 2015; Aarts et al., 2017).

Until recently, it was believed that urine under normal conditions was sterile. This has now been challenged by sensitive PCR-based techniques including 16S rRNA gene sequencing and expanded quantitative urine culture (EQUC). Studies are now emerging, investigating the urinary microbiota in various patient groups and healthy participants, showing that urine contains a plethora of bacteria with yet unknown function (Fouts et al., 2012; Wolfe et al., 2012; Hilt et al., 2014; Pearce et al., 2015; Thomas-White et al., 2016; Price et al., 2020). The urinary microbiota of healthy adult women (Siddiqui et al., 2011; Fouts et al., 2012; Wolfe et al., 2012; Lewis et al., 2013; Hilt et al., 2014; Pearce et al., 2014; Karstens et al., 2016; Thomas-White et al., 2016; Abernethy et al., 2017; Gottschick et al., 2017; Price et al., 2020) and men (Nelson et al., 2010, 2012; Dong et al., 2011; Fouts et al., 2012; Lewis et al., 2013; Gottschick et al., 2017; Bajic et al., 2020) have been investigated in a number of studies, whereas for children, only two studies have investigated the urinary microbiota of young children (Kassiri et al., 2019; Kinneman et al., 2020). The urinary microbiota of healthy women is mainly dominated by the genera *Lactobacillus, Gardnerella, Streptococcus*, and *Staphylococcus* (Wolfe et al., 2012; Hilt et al., 2014; Pearce et al., 2014, 2015; Karstens et al., 2016; Thomas-White et al., 2016; Abernethy et al., 2017; Price et al., 2020), whereas that of men is less explored, but often contains high relative abundance of *Lactobacillus, Staphylococcus, Streptococcus*, and *Corynebacterium* (Nelson et al., 2010, 2012; Dong et al., 2011; Fouts et al., 2012; Gottschick et al., 2017; Bajic et al., 2020). Importantly, sampling method can affect microbiota composition, with voided urine samples often being contaminated by vulvo-vaginal bacteria in females (Wolfe et al., 2012), and skin bacteria in both genders (Bajic et al., 2020). Despite these disadvantages, self-collection of voided urine samples are still highly valuable for community studies due to the invasive and specialized nature of catheterization (Price et al., 2020). It is assumed that the bacteria residing in the urinary tract can contribute to the health of the lower urinary tract (Brubaker and Wolfe, 2015; Abernethy et al., 2017). Notably, several studies have recently documented that an alteration in urinary microbiota correlates to diseases in the lower urinary tract, including urgency urinary incontinence (Pearce et al., 2015; Karstens et al., 2016; Thomas-White et al., 2016), urinary tract infections (Nienhouse et al., 2014; Kinneman et al., 2020), and other lower tract urinary symptoms (Fok et al., 2018) for women. More knowledge on the urinary microbiota may therefore help us to understand the etiology behind diseases of the lower urinary tract.

Despite the growing interest for urinary microbiota research, it appears that the methodologies and study designs, used in different studies, are highly heterogeneous (Jung et al., 2019; Bajic et al., 2020; Price et al., 2020), which makes it difficult to interpret and compare observed findings. Several protocol optimization studies have been conducted on fecal samples, providing valuable guidelines on how to store and process samples for gut microbiota studies (Wu et al., 2010; Mackenzie et al., 2015; Kia et al., 2016; Costea et al., 2017; Bundgaard-Nielsen et al., 2018; Panek et al., 2018). Importantly, the chemical content and structure of urine is very different from feces, leaving urinary microbiota research as an unexplored field regarding protocol recommendations. In fact, only a few studies have investigated the technical and methodological aspects of urine microbiota research (Dong et al., 2011; Wolfe et al., 2012; El Bali et al., 2014; Jung et al., 2019). These mainly focused on the urine collection method including suprapubic aspiration, clean-catch voided urine, or transurethral catheterization sample collection. Studies comparing the impact of urine storage conditions or time dependent changes on bacterial content are scarce. However, one study did investigate how different temperatures and use of a stabilization buffer could affect the urinary microbiota in healthy women (Jung et al., 2019). Jung et al. (2019) reported, that storage at room temperature significantly impacted the voided urinary microbiota, although the use of the preserving buffer AssayAssure® could alleviate this issue. Furthermore, a recent study by Price et al. (2020) demonstrated that the female urinary microbiota was affected by the menstrual cycle and sexual activity, indicating that time of urine collection may impact studies in women. Conversely, the stability of the voided urinary microbiota in children and adult males is still poorly understood.

We aimed to determine if different storage temperatures could influence voided urinary microbiota composition in healthy women. Furthermore, we also investigated if the composition remained stable throughout the day or between two different days in healthy women, men, and children.

## Methods

### Study Participants and Urine Collection

We included 24 healthy volunteers for this study. Study participants were free of bladder symptoms, based on self-reporting, and did not use any antibiotics within 3 months prior to participation in the study. In cases where medicine or hormonal contraceptives were used, these should be taken within the same period on all study days. Use of non-prescription painkillers was not accepted for up to 24 h prior to urine collection. In addition, the study participants were instructed to avoid urine collection during menstruation, and pregnant women were not included. The identity of all donors was anonymous, and no personal data were registered, besides sex, and age. For study part one, 5 women were recruited, and for study part two, 19 participants were recruited encompassing 5 men (18–50 years), 4 women (18–50 years), 5 boys (5–10 years), and 5 girls (5–10 years). Oral consent was obtained from all study participants, or from parents or other legal guardians if participants were below 18 years of age. The Regional Ethical Committee of Northern Denmark reviewed the study protocol. Since no personal information were collected from study participants and no intervention was performed, the Ethical Committee judged that no further approval was required.

**Figure 1:**
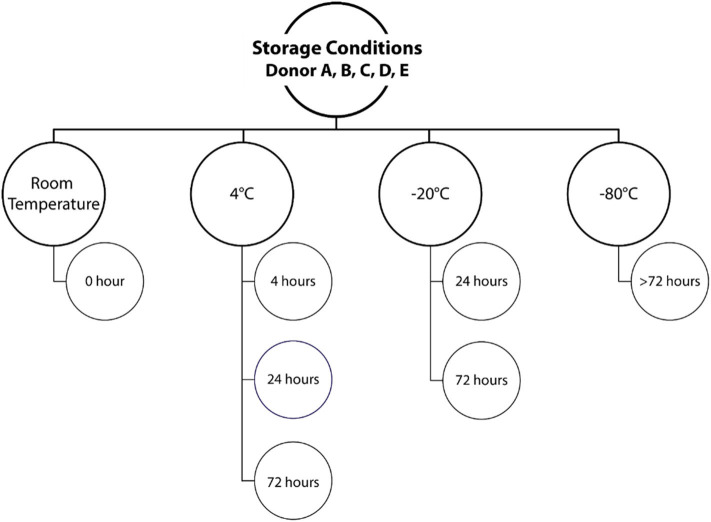
Urine storage conditions. Voided urine samples from each of the five study participants were either processed directly (room temperature, 0 h) or stored at different temperatures for different time periods. Duplicate samples were included for each storage condition.

For study part one, investigating the effects of different storage temperatures, voided urine was collected at the laboratory by the “clean catch” method. Briefly, the participants were asked to clean themselves around the urethra using a sterile wipe, and the first small amount of urine was urinated into the toilet bowl. Midstream urine was then collected into a specially designed cup. Samples were immediately aliquoted in tubes with 10 mL urine and transferred to the specified storage conditions, as summarized in Figure 1, or subjected directly to DNA extraction (RT sample). All conditions were tested in duplicates (two aliquots from each urine sample). For samples stored at −20°C, a freezer corresponding to a domestic freezer was used to mimic a home collection situation. All samples were finally collectively stored at −80°C, to rule out bias due to differences in low temperature exposure, until further processing.

For study part two on daily or day-to-day variations, the participants collected midstream urine at home by the clean catch method, and urine samples were immediately transferred to −20°C domestic freezers. All participants delivered two first morning voided urine samples (weekday and weekend) and two evening samples (weekday and weekend) and likewise, collections on a weekday or weekend day were represented by two independent samples (morning and evening). This gave rise to two independent samples per time point from each participant. Each independent sample was furthermore divided into two aliquots for duplicate DNA purification. Children were assisted by a parent to ensure correct sampling. Samples were transported on ice to the laboratory within 24 h. Upon arrival to the laboratory, the samples were stored at −80°C until further processing.

### DNA Purification

Bacterial DNA was isolated from 10 mL of urine using the QIAamp Viral RNA Mini Kit (Qiagen) according to manufacturer's recommendations. Prior to DNA extraction, urine samples were centrifuged at 3,000 × g for 20 min. Pellets were resuspended in PBS, lysis buffer added, and a bead beating step was included using the TissueLyser LT (Qiagen) for 2 min at 30 Hz with a 5 mm stainless steel bead. DNA yield was measured by the NanoDrop™ Lite Spectrophotometer (Thermo Fisher Scientific) or by fluorometric quantification using the Qubit 4 Fluorometer (Thermo Fisher Scientific) together with the Qubit dsDNA HS Assay Kit (Thermo Fisher Scientific).

### 16S rRNA Gene Sequencing

Bacterial 16S rRNA gene sequencing targeting the V4 region, was performed by DNAsense (Denmark), and followed a modified version of an Illumina protocol (Caporaso et al., 2012), as described by Albertsen et al. (2015), with an initial amplicon PCR. Due to upgrades in primer design during the experiment, different versions of reverse primers targeting the V4 region of the 16S rRNA gene were utilized in the different parts of this study. For the study on storage conditions, the following primer sequences were utilized (Forward: 5′-GTGCCAGCMGCCGCGGTAA-3′, reverse: GGACTAC**H**VGGGTWTCTAAT), while for the study investigating variations between evening and morning and day-to-day, a slightly modified reverse primer was used (5′-GGACTAC**N**VGGGTWTCTAAT-3′). Samples were pooled and sequencing was performed on a MISeq (Illumina, USA), as previously described (Caporaso et al., 2012). To measure error rate during sequencing and batch effects, a 20% PhiX control library was added. As negative control, nuclease-free water was used, while a complex sample obtained from an anaerobic digester system was utilized as a positive control.

### Bioinformatics and Statistics

Quality of sequencing reads was analyzed using FastQC (Babraham Bioinformatics, UK). Forward reads were quality trimmed using Trimmomatic v 0.32 (Bolger et al., 2014) to produce reads with a Phred score of at least 20 and a length of 250 bp. Subsequent bioinformatics followed the UPARSE workflow (Edgar, 2013) to remove chimeras, cluster operational taxonomic units (OTUs) based on 97% identity and assign taxonomy using the RDP classifier as previously described (Bundgaard-Nielsen et al., 2018). Data analysis was performed in R version 4.0.0 (R Development Core Team, 2019) through the Rstudio IDE (http://www.rstudio.com/) using the ampvis2 package v.2.6.0 (Albertsen et al., 2015), as well as Microsoft Office Excel 2013. Alpha-diversity was determined using OTU richness and Shannon Diversity Index, while beta-diversity was determined using principal component analysis (PCA) of variance in Hellinger transformed OTU abundances and heat maps displaying the most commonly found OTUs. For continuous data, distribution was tested using Shapiro-Wilks test while variance was tested using Bartlett's test. Normal distributed data was expressed by mean values and analyzed using students *t*-test (for intrapersonal variations at different timepoints) or ANOVA followed by Tukey's HSD *post-hoc* test (differences across storage groups), while similarly, non-parametric data was expressed as median values and analyzed using Wilcoxon-Mann-Whitney test or Kruskall-Wallis Test followed by Dunn's *post-hoc* test. Differences were considered statistically significant for *p* < 0.05.

## Results

### Different Storage Conditions Do Not Critically Affect Bacterial Composition

**Figure 2:**
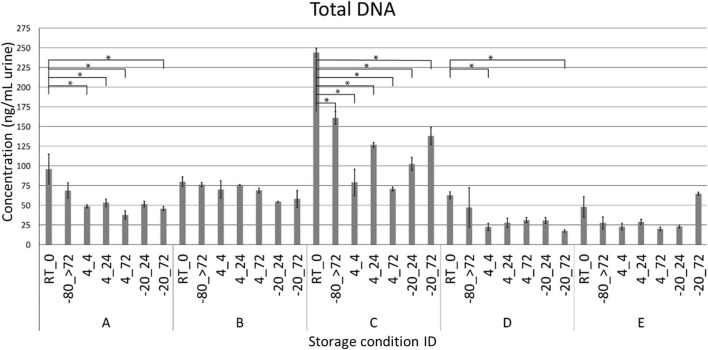
DNA yield. Quantity of total DNA purified from voided urine shown for each donor (A–E) and storage condition. Each sample is named as “temperature_duration.” *P < 0.05. RT = room temperature, >72 = sample are stored for at least 72 h.

Due to the risk of DNA degradation or bacterial growth, the ideal sampling strategy for voided urinary microbiota analyses would be to purify DNA immediately following delivery, or to transfer the urine samples directly to −80°C or colder. This is however not always possible or practical in a clinical setting, or when utilizing self-sampling at the home of the study participants. We therefore tested if storage of urine at different sub-optimal temperatures, altered the microbiota composition compared to a freshly processed sample. For this purpose, urine was collected from five healthy donors. Each urine sample was subsequently divided and stored according to one of the seven combinations of temperatures and times (Figure 1). Since each condition was tested in duplicate experiments, we reached a total of 70 samples. After the allocated storage period, total DNA was purified, and DNA concentrations obtained ranged from 16.2 to 248.0 ng/mL urine with minor variations within individual donors (Figure 2). With the exception of donor E, the highest DNA concentration was observed in the freshly processed sample, followed by samples stored directly at −80°C.

**Figure 3:**
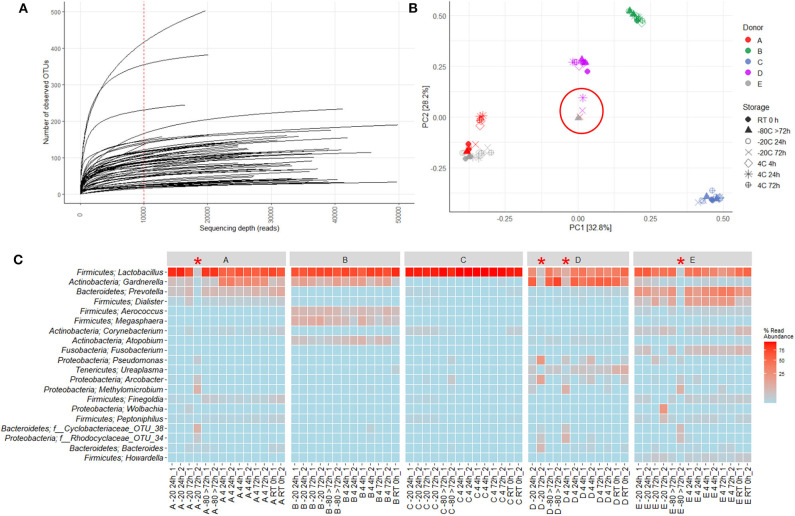
Quality control used for removing low quality samples. 16S rRNA gene sequencing of the V4 region gave rise to 2,097,325 reads (median 29,334, range 1,299–132,881 reads per sample). 933 unique OTUs were identified (median 106, range 33–502 OTUs per sample), with taxonomy assigned at the phylum level for 96.1% of OTUs and genus level for 53.2%. **(A)** Rarefaction Curve showing number of unique OTUs generated based on quantity of reads. The red dotted line indicates cutoff value of 10.000 reads. Three samples were excluded due to values below cutoff. **(B)** PCA based on Hellinger transformed OTU abundances. **(C)** Heatmap depicting the 20 most common OTUs for each separate duplicate. Each name consists of phylum followed by genus name. If no genus could be identified, the best taxonomic assignment is listed. The heatmap and PCA were used to identify potential outliers. The following four samples were excluded: A −20 72 h_2, D −20 72 h_2, D 4 24 h_1, and E −80 >72 h_2. These have been circled in **(B)** and marked with a red * in **(C)**.

**Figure 4:**
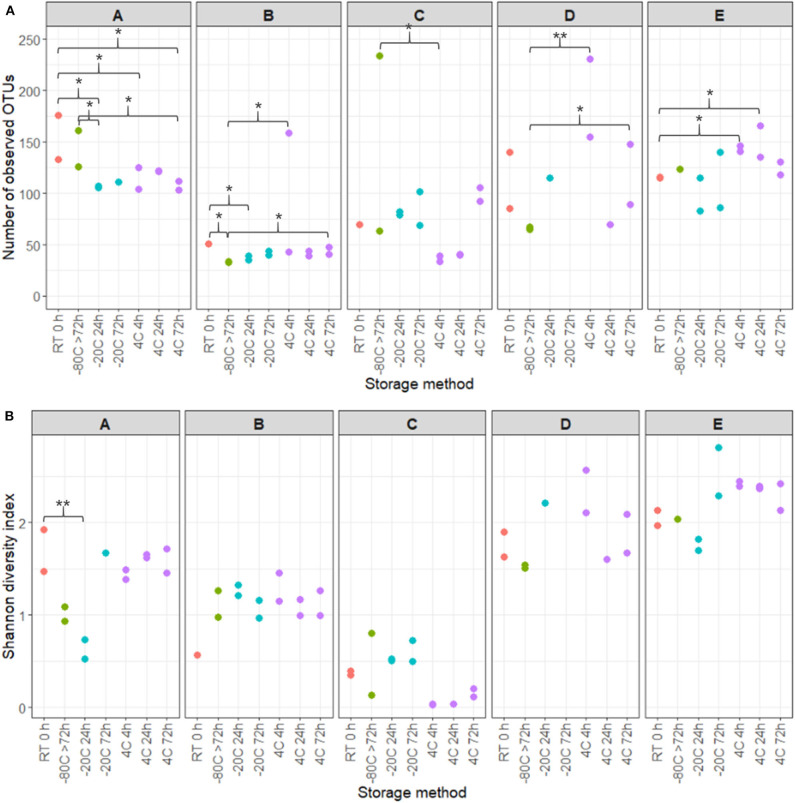
Effects of storage temperature and duration on voided urinary microbiota alpha diversity. **(A)** OTU richness showing numbers of unique OTUs observed in different storage conditions and durations. **(B)** Shannon Diversity Index visualizing differences and similarities in diversity of OTU composition within samples. Colors specify differing storage temperatures. Stars indicates statistics compared to RT and −80°C samples, with *p < 0.05, **p < 0.01.

**Figure 5:**
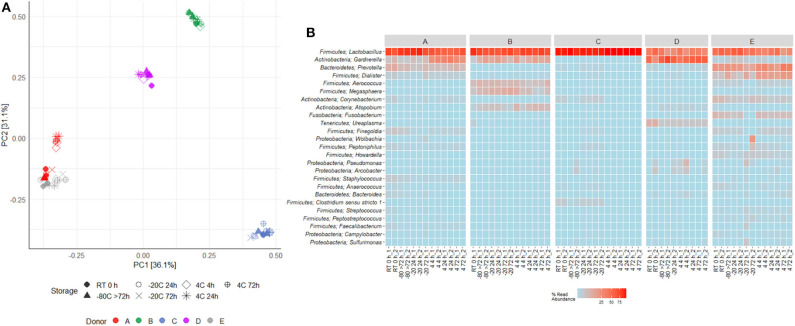
Effects of storage temperature and duration on voided urinary microbiota beta-diversity. **(A)** Clustering of samples based on PCA of Hellinger transformed OTU abundances. **(B)** Heat map depicting the 25 most common OTUs in duplicates in different storage conditions for each donor (A–E). Each name consists of phylum followed by genus name. If no genus could be identified, the best taxonomic assignment is listed.

16S rRNA gene sequencing of the V4 region resulted in a total of 2,097,325 reads (median 29,334, range 1,299–132,881 reads per sample). 933 unique OTUs were identified (median 106, range 33–502 OTUs per sample), with taxonomy assigned on the phylum level for 96.1% of OTUs and genus level for 53.2%. A rarefaction curve was generated and used to deselect samples that did not adequately cover all unique OTUs and therefore showed insufficient sequencing coverage (Figure 3A). Consequently, three samples were removed. Four samples were furthermore discarded based on poor duplicate comparison (Figures 3B,C), possibly caused by background contamination or high bacterial diversity in low-biomass samples. This led to a total of 63 samples being included in the following analyses. Alpha diversity metrics indicated that the microbiota profile was generally preserved when samples were immediately frozen at −80°C (Figures 4A,B). However, storage of samples at 4°C appeared to be suboptimal since the OTU richness for these were significantly different compared to freshly processed samples or samples stored directly at −80°C. In two cases (donors A and B, *p* < 0.05) the OTU richness differed between samples stored at −20°C and either freshly processed or −80°C samples. For Shannon diversity, however, the only difference observed was for donor A between freshly processed samples and samples stored at −20°C (*p* < 0.001, Figure 4B), indicating that the variation induced by 4°C only had a minor impact on overall diversity. In line with this, when looking at beta diversity, apart from samples stored at 4°C for donor A, variations between storage conditions were minor compared to interpersonal variations (Figure 5A). Compared to freshly processed samples, the relative abundance of the genera *Gardnerella* in donor A and *Dialister* in Donor E, was increased for samples stored at 4°C (Figure 5B). Overall, these results indicate that storage at −80°C can safely maintain the bacterial composition of voided urine samples. If conditions do not allow immediate processing or freezing at −80°C, as is often the case in studies utilizing home-sampling, storage at −20°C can effectively maintain bacterial composition. Storage at 4°C may impact composition of low abundance species, and thus, if possible, should be avoided.

### Voided Urinary Microbiota Composition Is Independent of Daily and Day-to-Day Variation

First morning urine is often more concentrated than subsequent urine samples throughout the day, while differences in daily routines (e.g., sleep rhythm, diet, sexual activity, or exercise) may introduce variations during the day (Price et al., 2020). We therefore speculated that morning urine could contain higher bacterial loads, and possibly a different bacterial composition than urine collected in the evening. To test this hypothesis, we compared voided urine samples collected in the morning and evening on two independent days from 19 healthy donors (4 women, 5 men, 5 girls, and 5 boys). Following DNA extraction, the resulting DNA yield ranged from <2.00 to 218.25 ng per mL urine. Importantly, DNA yield did not differ based on within day or day-to-day (data not shown).

Due to the collection method being performed under less controlled conditions (self-sampling by study participants), we expected a higher risk of contamination. In order to avoid false positive samples, we excluded samples that yielded less DNA following the initial PCR amplification for library preparation, compared to the negative controls (0.0538 ng/μL). Fifty-four of the original 152 samples were below cut-off levels for 1st PCR library amplification. Deselected samples were distributed unevenly as none were from women, 16 (40%) from girls, 26 (65%) from men, and 12 (30%) from boys. The remaining 98 samples (representing 17 participants: 4 women, 4 girls, 4 men, and 5 boys) were available for microbiota comparisons. These all showed good sequencing coverage based on a rarefaction curve (data not shown). 16S rRNA gene sequencing resulted in a total of 5,575,050 reads (median 52,232, range 7,529–122,101 reads per sample) and 2,538 unique OTUs (median 216, range 109–469 OTUs per sample) were identified. 93.1% were assigned to the phylum level and 50.9% to the genus level.

**Figure 6:**
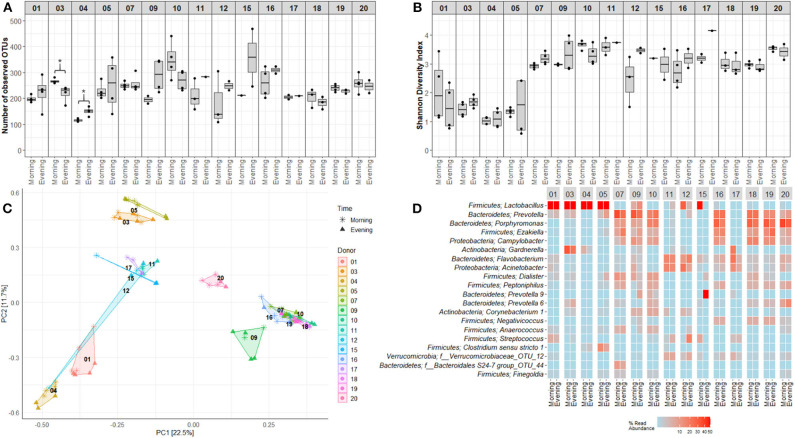
Within day variations in voided urinary microbial alpha and beta diversity. Visualization of variations and similarities between voided urine samples collected morning and evening within each study participant. **(A)** OTU richness showing numbers of unique OTUs in samples obtained morning and evening. **(B)** Shannon Diversity Index visualizing differences and similarities in diversity of OTU. **(C)** Distribution of samples collected mornings and evenings, using PCA of Hellinger transformed OTU abundances. Colors and numbers indicate study participant while point shape indicate collection time point. **(D)** Heat maps depicting the 20 most common OTUs in samples collected mornings and evenings. Each name consists of phylum followed by genus name. If no genus could be identified, the best taxonomic assignment is listed. For this part of the study, two donors, one girl (donor 8) and one man (donor 13), were excluded, since a complete set of matching evening-morning samples were lacking (due to quality control measures). Donors 01–05 were adult women (18–50 years), 06–10 girls (5–10 years), 11–15 adult men (18–50 years), and 16–20 were boys (5–10 years). *p < 0.05.

**Figure 7:**
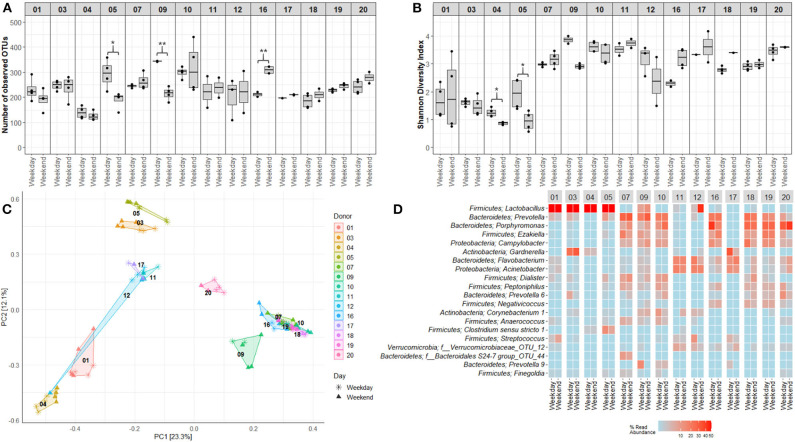
Variations in voided urinary microbiota alpha and beta diversity between weekdays and weekends. **(A)** OTU richness showing numbers of unique OTUs in samples obtained at different days. **(B)** Shannon Diversity Index visualizing differences and similarities in diversity of OTUs. **(C)** Distribution of samples collected at different days, using PCA of Hellinger transformed OTU abundances. Colors and numbers indicate study participant while point shape indicate collection day. **(D)** Heat maps depicting the 20 most common OTUs at different collection days. Each name consists of phylum followed by genus name. If no genus could be identified, the best taxonomic assignment is listed. For this part of the study, three donors, one girl (donor 8) and two men (donors 13 and 15), were excluded since a complete set of matching weekday-weekend samples were lacking. Donors 01–05 were adult women (18–50 years), 06–10 girls (5–10 years), 11–15 adult men (18–50 years), and 16–20 were boys (5–10 years). *p < 0.05, **p < 0.01.

Mapping of microbiota composition by 16S rRNA gene sequencing indicated that for a few study participants, the alpha diversity of voided urinary microbiota differed between morning and evening (Figure 6) and weekday and weekend (Figure 7), although these variations were minor, and the direction of change was inconsistent. For beta diversity, we observed that apart from the adult male participants 12 and 15, voided urine samples maintained similar bacterial compositions regardless of collection time point (Figures 6C,D, 7C,D). Intriguingly, while most samples clustered together with samples belonging to the same age and gender group (women, men, boys, and girls), one adult male sample taken on a weekend morning, clustered together with samples from adult women (donor 12, Figures 6C,D). This sample, together with morning samples from donor 15, had an increased relative abundance of *Lactobacillus*.

## Discussion

One of the great challenges, when performing microbiota studies on biological specimens, is always the risk of introducing bias due to methodological vulnerabilities. Studies investigating potential pitfalls are therefore essential to identify confounding factors. Here we show that the urinary microbiota is remarkably stable under different storage conditions and with respect to time.

While freezing samples at −80°C is considered gold standard for microbiota research (Bundgaard-Nielsen et al., 2018; Cumpanas et al., 2020), it is not always practical in a clinical setting as self-sampling by patients often requires samples to be stored short-term at the patients homes. We here report that the microbiota compositions of urine samples were maintained in all stored conditions for up to 72 h, except for samples stored at 4°C where we observed variations in OTU richness and DNA yield but not in Shannon diversity. Furthermore, samples from individual donors clustered together regardless of storage temperature and collection time, with the only exception being donor A where the 4°C samples clustered apart from the other samples. This is supported by a previous study showing that storage of urine samples at −20 or 4°C, maintained bacterial composition for up to 4 days (Jung et al., 2019). This resilience to relatively low temperatures allows collection and storage of urine samples at locations at distance from laboratory facilities. Previous studies have attempted to improve storage of urine samples through the use of storage buffers, like AssayAssure® (Kramer et al., 2018; Jung et al., 2019) or boric acid containing preservatives (Price et al., 2020). While promising for storage at suboptimal temperatures, like room temperature, the potential impact of these storage buffers on downstream laboratory procedures needs to be clarified.

The impact of sampling time on urine microbiota composition is still unclear. Importantly, we found that the voided urinary microbiota composition remained stable across different time points on a short-term basis. This is in contrast to a recent study by Price et al. (2020) that showed that the urinary microbiota of asymptomatic women, underwent recurrent shifts between different urotypes over time. Interestingly, in two men we observed inconsistency in the relative abundance of *Lactobacillus* between morning/evening and weekday/weekend samples. This may represent normal fluctuations in microbiota compositions or contamination with vaginal microbiota through intercourse prior to urine sampling, as suggested by Nelson et al. (2012). We did not collect data on sexual activity of the participants, which may be considered a potential confounding factor.

One obstacle, when investigating the urinary microbiota, is that only low amounts of bacterial DNA can be extracted from voided urine. A study by El Bali et al. (2014) reported that storage temperature had a major impact on DNA output levels. Importantly, they showed that storage at −20°C resulted in dramatically lower DNA yields compared to storage at −80°C or samples that were immediately processed. We did not observe the same level of DNA loss in samples stored at −20°C, which may be explained by the longer storage time (15 days) used by El Bali et al. Storage at −20°C for more than 72 h might therefore compromise urinary DNA integrity.

For samples collected in the home of participants, we observed that a relatively high number of these were excluded due to low DNA yield following the initial PCR during library preparation. Cutoffs based on low DNA or sequencing reads has previously been reported, and it appear to be essential for studies investigating microbiota of the low biomass voided urine samples (Pearce et al., 2015; Thomas-White et al., 2016; Bajic et al., 2020).

This study depended on self-sampling by the study participants in their homes and are thus performed in a less controlled environment. We show that the urinary microbiota is stable despite the use of home sampling which is important considering that several clinical or population-based studies cannot be performed in a laboratory or hospital environment. Voided urine samples are often contaminated by vulvo-vaginal bacteria in women (Wolfe et al., 2012), and skin bacteria in men (Bajic et al., 2020). This can be circumvented by the use of catheter, although this may not be available, or even beneficial (Pearce et al., 2014; Price et al., 2020). As a result, there is a need to investigate the resilience and dynamics of the voided urinary microbiota.

Our study suffers from certain limitations. First, we only evaluated the stability of the voided urinary microbiota over a short time period. Longer follow-up analyses would provide more information on bacterial fluctuations in men and children, adding to the observations on women in the study by Price et al. (2020). Another limitation is the large number of samples that were excluded due to first PCR DNA levels below cut-off values. Whether this fall-out of samples is due to technical issues, like DNA degradation or presence of PCR inhibitors, or due to biological differences in urinary bacterial loads between individuals, is unknown. The latter may be the case since we observed that a very high proportion of samples from men (65%) did not reach above cut-off levels, indicating that only very little bacterial DNA can be isolated from adult male voided urine samples. In comparison, none of the samples from women were discarded, 40% from girls, and 30% from boys. Our sample size is however too small to make any solid conclusions on age and gender differences in bacterial loads.

The strengths of this study are numerous. We have used a very systematic and structured approach with a relatively large number of samples from different participants. Importantly, when evaluating the day-to-day or daily variation, we included study participants of different gender and age. This was to consider whether there could be differences in time-dependent stability of different core microbiotas. Our data showed that, apart from two men, the urinary microbiota was stable regardless of gender and age. Finally, we take into account that very low levels of bacterial contamination may result in false positive samples, leading to misinterpretation of true microbiota profiles.

## Conclusion

In conclusion, we showed that the voided urinary microbiota is stable over time, and that sub-optimal temperatures for urine storage may be used, however care should be taken if using storage at 4°C. We recommend, however, that samples be transferred to −80°C as quickly as possible after collection, to avoid loss of the already limited DNA in voided urine samples. In addition, we highly recommend that besides including important clinical parameters such as diagnoses and medication, it is important to consider choice of storage condition and to implement good negative controls and use of background cut-off levels. The latter is especially important when working with low biomass samples. Finally, we recommend studies investigating the effects of sexual activity on the microbiota composition in urine of men, to determine if this may be a confounding factor. We encourage further studies on the methodological and technical aspects of urinary microbiota research with the aim of providing strong evidence-based guidelines.

## Data Availability Statement

For study part one, investigating the effects of different urine storage methodologies, sequencing data are available upon request. For study part two, investigating the effects of sampling time, sequencing data has been deposited at the Sequence Read Archive (SRA, NCBI USA) with BioProject ID 606994, available at https://www.ncbi.nlm.nih.gov/bioproject/606994.

## Ethics Statement

Ethical approval was not provided for this study on human participants because The Regional Ethical Committee of Northern Denmark reviewed the study protocol. Since no personal information were collected from study participants and no intervention was performed, the Ethical Committee judged that no further approval was required. Written informed consent to participate in this study was provided by the participants' legal guardian/next of kin.

## Author Contributions

SS, SH, NA, and CB-N designed this study. NA, YI, and AM performed the experiments. SS, CB-N, NA, YI, and AM prepared the manuscript. All authors read and approved the final manuscript, participated in data analysis, and finalized the manuscript.

## Conflict of Interest

The authors declare that the research was conducted in the absence of any commercial or financial relationships that could be construed as a potential conflict of interest.

## Supplementary Material

Supplementary Table 1 16S rRNA gene sequencing metadata. Information describing the samples used for investigating storage.

Supplementary Table 2 16S rRNA gene sequencing OTUtable. OTU table documenting numbers of different OTUs generated during the 16S rRNA gene sequencing of the samples used for investigating storage.

Supplementary Table 3 16S rRNA gene sequencing metadata. Information describing the samples used for investigating bacterial variation across different time of day or between weekday or weekend.

Supplementary Table 4 16S rRNA gene sequencing OTUtable. OTU table documenting numbers of different OTUs generated during the 16S rRNA gene sequencing of the samples used for investigating bacterial variation across different time of day or between weekday or weekend.
